# HIV Prevention and Rehabilitation Models for Women Who Inject Drugs in Russia and Ukraine

**DOI:** 10.1155/2012/316871

**Published:** 2012-12-05

**Authors:** Roman Yorick, Halyna Skipalska, Svetlana Suvorova, Olga Sukovatova, Konstantin Zakharov, Sara Hodgdon

**Affiliations:** ^1^HealthRight International, Representative Office in the Russian Federation, 7 Lev Tolstoy Street, Office 701, St. Petersburg 197376, Russia; ^2^Doctors to Children, 7 Lev Tolstoy Street, Office 707, St. Petersburg 197376, Russia; ^3^HealthRight International, Representative Office in Ukraine, 3-7 Stanislavsky Street, Kyiv 01001, Ukraine; ^4^Ukrainian Foundation for Public Health, 3-7 Stanislavsky Street, Kyiv 01001, Ukraine; ^5^HealthRight International, 65 Broadway, 19th Floor, New York, NY 10006, USA

## Abstract

Women who inject drugs require gender-specific approaches to drug rehabilitation, modification of risk behaviors, and psychosocial adaptation. Improved outcomes have been demonstrated when the specific needs of women's subpopulations have been addressed. Special services for women include prenatal care, child care, women-only programs, supplemental workshops on women-focused topics, mental health services, and comprehensive programs that include several of the above components. To address the special needs of women injecting drug user (IDU) subpopulations, such as HIV-positive pregnant women and women with young children, recently released female prisoners, and street-involved girls and young women, HealthRight International and its local partners in Russia and Ukraine have developed innovative service models. This paper presents each of these models and discusses their effectiveness and implementation challenges specific to local contexts in Russia and Ukraine.

## 1. Introduction

The prevalence of injecting use of opioid substances among people 15–64 years of age is estimated at 0.9% in Ukraine and 2.29% in Russia [1]. There is no reliable data on gender distribution of IDUs in the region. Global trends suggest that drug use in general is more prevalent among boys and men than among girls and women [2]. Some data from the region demonstrate that women constitute between 20 and 30% of IDUs [3, 4]. The HIV epidemic in Russia has been closely linked with IDU: 56.2–61.3% of newly reported HIV infections in 2008–2011 were attributed to IDUs [5]. In Ukraine, heterosexual sex has been the predominant route of HIV transmission since 2008, but drug use is still a key force in the epidemic, with 38.4% of new infections occurring in IDUs in 2011 [6]. The midestimates of HIV prevalence in IDUs in the region are close to 40% [7].

Female sub-populations with the highest prevalence of injecting drug use and HIV in the region include commercial sex workers (CSWs) and those who engage in transactional sex, street-involved girls and young women, and prisoners. These groups are highly intertwined. Among CSWs, 18–70% inject drugs [8–10]; among IDUs, some 40% report transactional sex [11]. Among street children and youth, approximately one-third are female; 20–50% have experience of injecting drug use, and 5–10% report transactional sex [12–14]. Women constitute 5.3% and 8.2% of the prison populations in Russia and Ukraine, or 60 and 8 thousand women prisoners, respectively [15]. There are no reliable national data on the proportion of IDUs among prisoners in the region. In some regions of Russia and Ukraine, the proportion of IDUs among inmates is greater than 10%, HIV prevalence in prisons is greater than 10%, and HIV prevalence among inmates who have used injecting drugs is close to 50% [16]. Evidence from other parts of the world suggests that, compared to men, a larger proportion of women in prisons is convicted for drug-related crimes, and between 20 and 70 percent of women in prisons are drug-dependent [17–20].

A number of studies conducted within the last 20 years have addressed gender differences in the needs, experiences and rehabilitation approaches for drug users [21–23]. In particular, women start using drugs later than men, and their addiction progresses much faster. Women's substance abuse practices are formed by their male partners. Women demonstrate a much higher prevalence of psychiatric conditions, such as depression, that coexist with and predate substance abuse [24]. Women are less likely than men to access drug rehabilitation treatment, and for this treatment to be effective it has to address the needs of specific subgroups of women. Special services for women include prenatal care, child care, women-only programs, supplemental workshops on women-focused topics, mental health services, and comprehensive programs that include several of the above components [25]. 

To address the needs of drug-using women in Russia and Ukraine, the global health and human rights organization HealthRight International and its local implementing partners developed a model to assist HIV-positive pregnant women and women with young children (MAMA+), a model for psychosocial adaptation of recently released female prisoners, and comprehensive services to assist street-involved girls and young women.

Medication-assisted treatment for women IDUs is an important asset to psychosocial rehabilitation services. As most IDUs in Russia and Ukraine are opioid-dependent, they can benefit from opioid substitution therapy (OST). In Russia, however, OST is not permitted, with methadone being on the list of illegal substances and buprenorphine not approved for OST [26]. The only other medication option for opioid-dependent IDUs is the opioid antagonist naltrexone. Despite a number of studies in Russia demonstrating its effectiveness in preventing relapse in heroin users [27–31], naltrexone remains largely unavailable due to its prohibitive cost: a recommended nine-month treatment course costs over $6,000 [32]. In September 2012, naltrexone became available in St. Petersburg through a government-funded program to just 40 HIV-positive IDUs a year citywide to prevent the spread of HIV [33].

In Ukraine, OST with buprenorphine has been available since 2004, with methadone added in 2008 [30]. Early OST rollout data showed that women, particularly pregnant women and women with young children, were not accessing OST in proportion to their presence in the IDU population [4, 34]. A number of restrictions apply to OST access in Ukraine raising the entry threshold for potential participants. Among others, these limitations include the requirement to produce an identification document, which many IDUs do not have, or present a certificate of two unsuccessful attempts of nonmedicated rehabilitation within the last year [35]. The MAMA+ for IDUs model in Kyiv worked to overcome these shortfalls by incorporating OST as an important component of psychosocial rehabilitation of women IDUs [33, 35]. 

## 2. MAMA+: Comprehensive Assistance to **** HIV-Positive Pregnant Women and Women**** with Young Children

The MAMA+ model was initially developed in 2004 in St. Petersburg, Russia, to prevent child abandonment by HIV-positive mothers. That year, the rate of child abandonment by HIV-positive women in St. Petersburg was 11.7%, ranging from 5.5% in noninjectors to 15.6% in IDUs [36]. HealthRight, the St. Petersburg-based NGO Doctors to Children (DTC), and government Centers of Social Services for Families and Children in three districts of the city implemented the MAMA+ model to deliver psychosocial services for HIV-positive women at high risk of child abandonment. Other partners include the city infectious disease hospital where women with known HIV-positive status deliver and the only two maternity hospitals in the city that admit women for delivery who have not previously accessed prenatal care. These two high-risk maternity hospitals provide women in labor with counseling and rapid testing for HIV. This allows for catchment of all HIV-positive pregnant women perinatally. Prenatal clinics or women's consultancies and NGOs serving IDUs and people living with HIV were later added to the referral network to allow for earlier identification of HIV-positive pregnant women. Risk factors for abandonment include expressed intention to abandon, injecting drug use, depression, no stable housing, family violence, and lack of support from family members. Initial screening is conducted by healthcare staff who refer at-risk women to professional counselors. After additional screening, motivational interviewing, and crisis psychological counseling, the counselor asks the woman to sign an informed consent for services.

**Figure 1:**
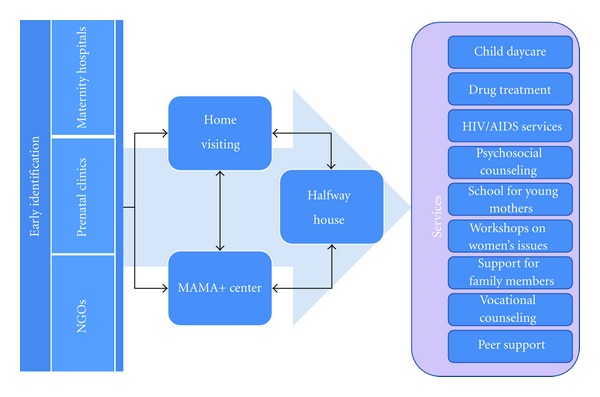
MAMA+ model.

**Figure 2:**
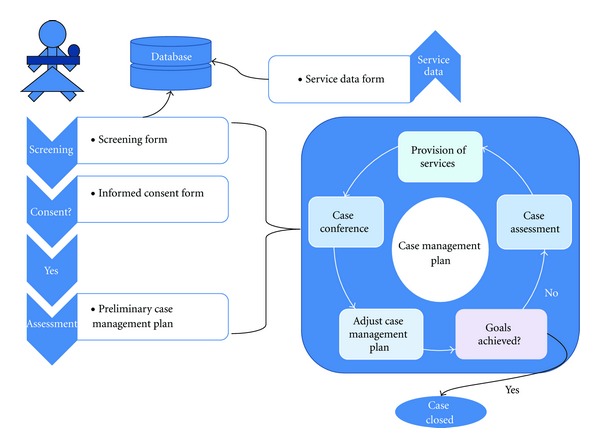
Case management protocol.

The model is comprised of the following components (Figure 1): (1) home-visiting services; (2) child daycare at the MAMA+ center; (3) halfway house residential support for pregnant women and women with young children; (4) counseling and referral to government and community-based drug treatment programs; (5) social counseling, referral and escort to government institutions to process paperwork, apply for housing, receive government child allowance, and so forth; (6) school for young mothers on child care, parenting, and HIV-related issues in children; (7) workshops on women's issues, such as improving self-image and self-esteem, coping with family violence, and building independence; (8) workshops for family members on HIV, care and support for women and children affected by HIV, and substance codependence; (9) vocational counseling and job placement for women and family members; (10) peer support groups for women, their partners, and other family members; (11) peer counseling by former MAMA+ clients. This model is implemented by a multidisciplinary case management team comprised of at least one of each of the following professionals and supplemented by peer volunteers: social workers, psychologists, child development specialists, medical providers (nurse or nurse practitioner), and lawyers. All services are provided according to a multidisciplinary case management protocol (Figure 2), which has been documented and published in Russian and Ukrainian [37, 38].

In Russia, the MAMA+ model has been implemented in the cities of St. Petersburg and Yekaterinburg in partnership with NGOs and government Centers of Social Services for Families and Children. The network of government social service facilities has been the single most important factor contributing to the model's sustainability in Russia. Most components of the MAMA+ model have been institutionalized as part of government Centers of Social Services, with 437 government providers receiving training and supervision from HealthRight and DTC in 2007–2012. In 2005–2011, 808 HIV-positive women and their children and other family members received MAMA+ services in St. Petersburg, with a specific emphasis on drug-using women as most at-risk of child abandonment. The proportion of IDUs among HIV-positive pregnant women in St. Petersburg decreased from 62.3% in 2004 to 40.9% in 2008 [36]. At the same time, IDUs constituted approximately 80% of MAMA+ clients in St. Petersburg [37]. As a result, child abandonment by HIV-positive women decreased from 11.7% in 2004 to 6.0% in 2008, with the most significant decrease of the abandonment rate among injecting drug users, from 15.6% to 9.9% (*P* = 0.009) [36]. In 2010–2012, as a result of MAMA+ replication in the city of Yekaterinburg, child abandonment rates among HIV-positive women dropped from 6.3% in 2009 to 1.9% in 2011 (*P* = 0.0037) [39]. Most HIV-positive women who abandon their children, even with available MAMA+ services, are active IDUs who do not access prenatal care. They learn about their HIV status in the maternity hospital and leave the hospital within hours after delivery seeking their next dose of illicit drugs, as opioid substitution therapy (OST) is not available in Russia [40, 41].

In Ukraine, where MAMA+ was replicated in 2005 in the cities of Kyiv, Donetsk, and Simferopol, OST with buprenorphine has been available since 2004, with methadone added in 2008 [40]. Originally, only 25% of MAMA+ clients in Ukraine were IDUs, reflecting the national trends of the HIV epidemic [6, 42]. Injecting drug use, however, has been identified as the single most important risk factor for child abandonment by HIV-positive women in Ukraine [43]. Thus the MAMA+ model, which was implemented in partnership with the All-Ukrainian Network of People Living with HIV (AUN) and the Ukrainian Foundation for Public Health, was adapted for IDUs in Kyiv city. Services added to better meet the needs of IDUs included counseling by a drug-abuse physician (narcologist), referral to OST programs, and a peer support group for IDU pregnant women and women with young children [44, 45]. In 2008–2010, 100 female IDUs who were pregnant or had young children, as well as 139 of their family members received MAMA+ for IDUs services, and 14 women were linked with OST programs in Kyiv [46]. Unlike in Russia, where the model benefitted from government investment in a social service system, the main factor of model sustainability in Ukraine has been local NGOs and international donors. The AUN has disseminated the MAMA+ model across Ukraine beyond the original three cities and has continued to support MAMA+ for IDUs in Kyiv.

## 3. Psychosocial Adaptation of Recently Released Female Prisoners

To respond to the specific needs of female prisoners, such as HIV/AIDS, sexual and reproductive health concerns, drug use, child custody, family support, and other issues [17, 47], HealthRight, in partnership with DTC and regional social protection and penitentiary authorities, developed a model on psychosocial adaptation of recently released female prisoners. The model was implemented in 2010–2012 in a medium-security female penitentiary facility in the Leningrad region near St. Petersburg. Among 800–1,200 inmates, 67% are under the age of 35; 51% have children under 18; over 60% were sentenced for drug-related crimes, many of whom have experience of injecting drug use; 37% are HIV-positive; 45% of those who are positive require ARV treatment. 

**Figure 3:**
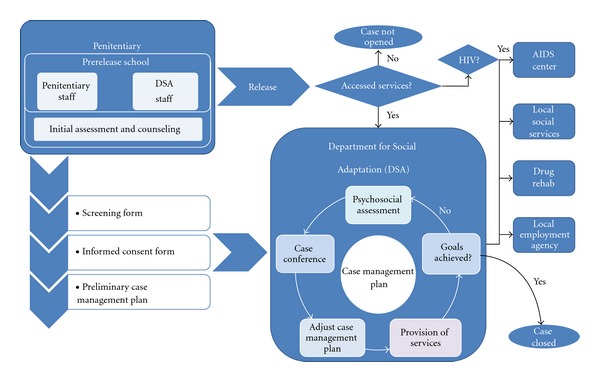
Psychosocial adaptation of recent female prisoners.

The model components include the following (Figure 3): (1) a specialized Department for Social Adaptation (DSA) of recently released female prisoners at the government Crisis Center for Women in St. Petersburg; (2) prerelease school at the penitentiary facility conducted by DSA staff; (3) individual psychosocial counseling at the penitentiary and after release; (4) group training activities on HIV prevention, drug abuse, sexual risks, and other risky behaviors before and after release; (5) laboratory monitoring of immune status, consultations by an infectious disease specialist, and access to ARV treatment for HIV-positive women in prison; (5) peer support groups for IDUs and HIV-positive women at the penitentiary and at the DSA upon release; (6) psychosocial support upon release to secure necessary documents, housing, employment, child custody, and so forth; (7) referral and support in accessing government and NGO-run drug rehabilitation programs and community-based twelve-step programs; (8) referral for AIDS Center services, ARV treatment, and support in treatment adherence; (9) psychosocial counseling and peer support groups for family members of prisoners. 

The team of providers is comprised of the team leader (head of the DSA), DSA social workers and psychologists, penitentiary counselors, and a DSA lawyer. All services are provided according to the case management and cross-referral protocol, which was approved by the regional social protection and penitentiary authorities and published in the form of methodological recommendations for social service providers and penitentiary staff [48].

Over two years, over 1,200 women received services in the penitentiary facility regardless of their HIV or IDU status, and 287 (or 24%) accessed services in St. Petersburg upon release. This access rate is comparable to the 30% rate of access to HIV services reported in the United States for HIV-positive recently released inmates [49, 50]. A key factor of success in linking former female prisoners to services is utilization of the same staff who established a rapport with inmates before release to provide services after release. Access to postrelease services is impeded by the fact that over 30% of the inmates at the facility in the Leningrad region are not from St. Petersburg and so do not seek services in the city upon release. Linking IDUs to residential drug treatment programs immediately upon release has been crucial in preventing relapse. Access to HIV treatment and care for female prisoners in Russia has been significantly impaired in 2011-2012 by the reform that transferred penitentiary healthcare to the responsibility of the civilian healthcare system, but failed to effectively link civilian providers with prison populations. 

## 4. Comprehensive Services for Street-Involved Girls and Young Women

**Figure 4:**
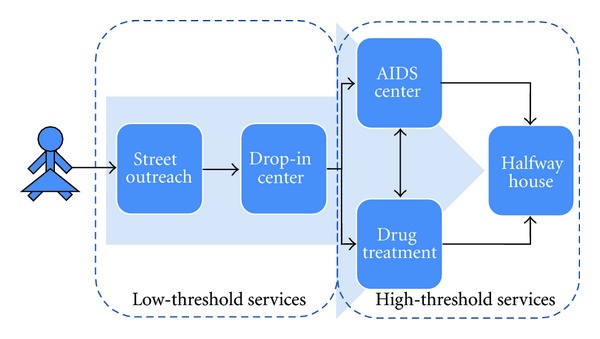
Comprehensive services for street-involved girls and young women.

HealthRight and the US Centers for Disease Control and Prevention conducted an HIV seroprevalence study among street youth in Ukraine in 2008, which demonstrated that girls are at a significantly higher risk of HIV, suggesting the need to implement services specifically targeting street-involved girls and young women [13]. In response, in 2010-2011 HealthRight in partnership with the Ukrainian Foundation for Public Health and the government Kyiv City Center of Social Services for Families, Children and Youth developed a model serving street-involved girls and young women. The model includes the following components (Figure 4): (1) street outreach by partner organizations providing psychosocial counseling to all street youth and referral for street-involved girls and young women to the project drop-in center (DIC); (2) low-threshold DIC where clients receive access to a safe space, shower, clean clothes, snacks, and case management services; (3) halfway house residential support for street-involved girls and young women (this component is currently in development); (4) psychosocial counseling for girls and young women on government paperwork, housing, education, employment, and other issues; (5) voluntary counseling and rapid testing for HIV, STIs and pregnancy at the DIC, and referral to specialized clinics; (6) behavior change communication intervention entitled STEPS to reduce HIV and other risks, which was developed specifically for street youth [51–54]; (7) training activities and workshops on women-related topics, such as gender-based violence, sexual and reproductive health, self-image and self-assurance; (8) peer support groups for girls and young women; (9) referral to drug abuse rehabilitation and OST, where appropriate; (10) services for male partners of DIC clients, including counseling, education, and group activities.

Services are provided according to a case management protocol to assist street youth, which was documented and published in Russian and Ukrainian [55–58]. The case management team includes the team leader, a medical provider (nurse), social workers, psychologists, and a lawyer. Since 2012, the DIC and its entire staff have been integrated into the government Kyiv City Center of Social Services. In 2010-2011, 759 girls and young women received services. Of them, 42% sleep on the streets, in basements, attics, or abandoned buildings; 13% are substance users; 39% have children of their own; 26% are HIV-positive [59]. 

This gender-sensitive model of services for street-involved girls and young women was developed from a coeducational model implemented earlier in Russia and Ukraine [54–57]. The gender-specific approach has proven successful in making services more attractive and accessible for female clients, providing counseling and group activities on woman-related topics, and addressing some specific needs of women, such as pregnancy and sexual health in a woman-friendly atmosphere. At the same time, it has proven unproductive to exclude male partners of DIC clients from counseling services and group activities, as street-involved girls and women are often dependent on men in their sexual, drug using, and other behaviors, as well as for protection and financial support. Coeducational service facilities are required to provide psychosocial rehabilitation and support to street youth, with special attention to gender-sensitive topics addressed in separate and mixed-sex groups.
